# Efficacy of a mechanism-based psychological intervention for persistent gastrointestinal symptoms in ulcerative colitis and irritable bowel syndrome: results of a three-arm randomised controlled trial (SOMA.GUT-RCT)

**DOI:** 10.1016/j.eclinm.2025.103663

**Published:** 2025-11-28

**Authors:** Kerstin Maehder, Luisa Peters, Sina Hübener, Anna Matysiak, Samuel Huber, Anne Toussaint, Antonia Zapf, Eik Vettorazzi, Philipp Weber, Katharina Stahlmann, Viola Andresen, Yvonne Nestoriuc, Ansgar W. Lohse, Bernd Löwe

**Affiliations:** a Department of Psychosomatic Medicine and Psychotherapy, University Medical Centre Hamburg-Eppendorf, Martinistr. 52, 20246 Hamburg, Germany; b I. Department of Medicine, University Medical Centre Hamburg-Eppendorf, Martinistr. 52, 20246 Hamburg, Germany; c Institute of Medical Biometry and Epidemiology, University Medical Centre Hamburg-Eppendorf, Martinistr. 52, 20246 Hamburg, Germany; d Medizinicum, Stephansplatz 3, 20354 Hamburg, Germany; e Department of Clinical Psychology and Psychotherapy, Helmut Schmidt University/University of the Federal Armed Forces, Holstenhofweg 85, 22043 Hamburg, Germany

**Keywords:** Irritable bowel syndrome, Ulcerative colitis, Randomised controlled trial, Psychotherapy, Persistent somatic symptoms, Persistent physical symptoms, Expectations, Anxiety

## Abstract

**Background:**

Irritable Bowel Syndrome (IBS) and Ulcerative Colitis (UC) are chronic gastrointestinal conditions with differing pathologies but overlapping symptoms and shared risk factors contributing to symptom persistence. Illness-related anxiety and dysfunctional symptom expectations constitute two empirically proven mechanisms of symptom persistence. Yet, the efficacy of a targeted psychological intervention focussing on these mechanisms remains untested. The SOMA.GUT trial tested whether such a mechanism-based intervention can reduce gastrointestinal symptom severity in individuals with IBS or UC.

**Methods:**

The SOMA.GUT trial was an investigator-initiated, three-arm randomised controlled trial with nationwide recruitment in Germany of adult patients with either IBS or UC and at least moderate gastrointestinal symptom severity (Irritable Bowel Syndrome—Severity Scoring System, IBS-SSS ≥175). Patients were randomly assigned (1:1:1) to one of three groups: to standard care (SC) alone; to a targeted psychological intervention addressing illness-related anxiety and dysfunctional symptom expectations (GUT.EXPECT + SC); or to a supportive intervention designed to give insights into non-specific treatment effects (GUT.SUPPORT + SC). Randomisation was stratified by gender and diagnosis. Both intervention arms comprised four therapist-guided online sessions. The primary outcome was change in IBS-SSS gastrointestinal symptom severity between baseline and three months in the intention-to-treat population. Key secondary outcomes, measured at six weeks, three, six, and 12 months, included the mechanisms targeted by the GUT.EXPECT intervention. This trial was registered (ISRCTN30800023) and has been completed.

**Findings:**

Between April 2022, and February 2024, 2099 patients were screened online for eligibility. Of the 240 patients included in the full analysis set (UC: 126, IBS: 114), 176 (73.3%) self-identified as female, 62 (25.8%) as male and two (0.8%) as diverse. Change in IBS-SSS gastrointestinal symptom severity at three months did not differ significantly between groups (global p = 0.83); SC: −50.4 (95% CI −70.7 to −30.1), GUT.SUPPORT + SC: −55.4 (−75.0 to −35.9) and GUT.EXPECT + SC: −59.4 (−79.4 to −39.4). However, in exploratory analyses, the GUT.EXPECT intervention group showed relevant improvements in illness-related anxiety and expectations about symptom coping at three months compared to SC only, as well as in IBS-SSS gastrointestinal symptom severity at 12 months. No differential treatment effects were observed for UC and IBS, and no intervention-related serious adverse events were reported in any group.

**Interpretation:**

While gastrointestinal symptom severity remained similar between the groups at three months, the targeted psychological variables improved, and the symptom improvement observed at 12 months might indicate a delayed effect of the mechanism-based intervention. These findings may support the relevance of the proposed biopsychosocial mechanisms and the potential of psychological interventions in gastrointestinal disorders. However, higher treatment intensity, and broader biopsychosocial targeting may be required to achieve more immediate and clinically meaningful symptom relief.

**Funding:**

10.13039/501100001659German Research Foundation (Deutsche Forschungsgemeinschaft, DFG, Project ID: 445297796).

**Research in context**

**Evidence before this study**

To identify clinical trials on psychological interventions for gastrointestinal symptom severity in both irritable bowel syndrome and inflammatory bowel disease, PubMed and MEDLINE databases were searched for studies published from January 1, 2000, to August 31, 2025. The search was performed before and after the trial with the following search terms: “irritable bowel syndrome”, “inflammatory bowel disease”, “ulcerative colitis”, “psychological intervention”, “cognitive-behavioural therapy”, “mechanism-based intervention”, and “gastrointestinal symptoms”. Only articles published in English or German were included. We also manually searched studies cited in systematic reviews, meta-analyses, or randomised controlled trials as well as main trial registries. While the evidence for psychological interventions in IBS indicates effectiveness on gastrointestinal symptoms particularly for cognitive-behavioural therapy and gut-directed hypnotherapy, evidence for the impact of psychological interventions on gastrointestinal symptoms in inflammatory bowel disease, particularly on ulcerative colitis, reveals only limited evidence with mixed results so far. Mechanism-based psychological targets that have the potential to improve gastrointestinal symptoms include illness-related anxiety and dysfunctional expectations. However, thus far, no studies tested these specific mechanisms. Therefore, clinical trials investigating mechanism-based interventions for gastrointestinal symptoms in chronic gastrointestinal conditions are warranted.

**Added value of this study**

The SOMA.GUT trial is the first randomised controlled trial to test a mechanism-based intervention targeting illness-related anxiety and dysfunctional symptom expectations (GUT.EXPECT) in order to reduce gastrointestinal symptom severity in patients with ulcerative colitis or irritable bowel syndrome. The assumed mechanisms of change are grounded in the biopsychosocial model of persistent physical symptoms, herein adapted to chronic gastrointestinal disorders, comparing both an inflammatory disease and a disorder of gut–brain interaction. No significant difference in change in gastrointestinal symptoms between baseline and 3-month follow-up was found between the three groups: standard care (SC) only, GUT.EXPECT + SC, and GUT.SUPPORT + SC — a non-specific supportive intervention designed to elucidate non-specific therapeutic effects. However, exploratory analyses of patient-relevant secondary outcomes including the targeted mechanisms, i.e., illness-related anxiety and dysfunctional expectations, showed a relevant improvement at three months in the GUT.EXPECT + SC group, indicating that the targeted mechanisms are modifiable. This effect on the targeted mechanisms in the GUT.EXPECT + SC group was followed by a more favourable development in gastrointestinal symptom severity at 12 months, suggesting a delayed effect on symptoms. Subgroup analyses showed that treatment effects were consistent across patients with irritable bowel syndrome and those with ulcerative colitis, indicating no evidence of differential efficacy between the two diagnostic groups. The intervention evaluation showed high patient satisfaction in both groups, and no treatment-associated adverse events occurred.

**Implications of all the available evidence**

The targeted mechanisms of illness-related anxiety and dysfunctional symptom expectations in irritable bowel syndrome and ulcerative colitis appear amenable to change by a mechanism-based psychological intervention. However, these changes did not result in an immediate reduction in gastrointestinal symptoms compared to standard care. Instead, a delayed yet relevant advantage of the GUT.EXPECT + SC intervention group became apparent over time, which might suggest a possible benefit of the mechanism-based intervention with regard to gastrointestinal symptoms. The potential modifiability of the psychological targets — and their assumed eventual impact on symptom severity — supports the relevance of the hypothesised mechanisms. To achieve earlier and more pronounced effects on gastrointestinal symptoms in irritable bowel syndrome and ulcerative colitis, a higher therapeutic dose and the targeting of additional and/or personalised biopsychosocial mechanisms may be required.

## Introduction

Irritable bowel syndrome (IBS) and ulcerative colitis (UC) are prevalent chronic conditions, affecting about 4% and 0.3% of the global population, respectively.^1^^,^^2^ Both share persistent physical symptoms, including abdominal pain and altered bowel habits. In IBS, these symptoms are a defining diagnostic feature, whereas in UC they occur during acute flares and persist in approximately 29% of patients even during remission.^3^^,^^4^ Despite their burden, treatment options for persistent physical symptoms in IBS and UC remain inadequate. Recent evidence-based biopsychosocial models have identified modifiable risk factors, such as dysfunctional expectations and illness-related anxiety, as central contributors to symptom persistence across diverse conditions.^5^^,^^6^ These factors are also highlighted in disease-specific models for IBS and UC.^7^ Yet, their therapeutic modification has not been tested in randomised trials. Given the symptom overlap and shared psychological risk factors, trials targeting these factors in IBS and UC offer a unique opportunity to uncover cross-disease and disease-specific mechanisms of symptom persistence.

For IBS, a large meta-analysis supports the efficacy of psychological interventions for global improvement and abdominal pain, particularly for cognitive-behavioural therapy (CBT) and gut-directed hypnotherapy.^8^ However, the mechanisms underlying symptom improvement remain poorly understood. Illness-related anxiety, dysfunctional expectations, and avoidance behaviours have been identified as potential therapeutic targets, while targeting stress has only shown limited benefits so far.^7^^,^^9^, ^10^, ^11^, ^12^ In addition, higher initial symptoms, dysfunctional cognitions such as pain catastrophising, and anxiety, as well as positive treatment expectations and illness-related self-efficacy beliefs have been linked to treatment response.^13^, ^14^, ^15^ Further studies are needed to clarify these putative mechanisms and their influence on psychosocial and somatic outcomes.

For inflammatory bowel disease (IBD), including UC, evidence for psychological interventions is less conclusive. Meta-analyses and a recent Cochrane review indicate short-term benefits for anxiety, depression, stress and quality of life, but minimal or no effects on markers of disease activity.^16^, ^17^, ^18^, ^19^ Nevertheless, bi-directional interactions between biological and psychosocial factors in IBD, including depression, anxiety and stress, are widely accepted.^20^^,^^21^ Psychological burden has been associated with IBS-like symptoms even in quiescent IBD and with long-term disease outcomes.^22^ Moreover, psychosocial factors such as depression, anxiety, negative symptom perceptions, all-or-nothing and avoidance behaviours have been linked to more severe symptoms profiles.^23^ A first study in Crohn's disease indicates an association of symptom and treatment expectations with subjective symptom improvement.^24^ Together, these findings underline the relevance of psychosocial factors, such as anxiety and expectations, for the course of IBD symptoms, while also pointing to critical gaps in understanding their therapeutic relevance.

While depression and stress have been targeted in treatments for IBD and IBS, illness-related anxiety and dysfunctional expectations have not yet been explored despite their therapeutic potential. The potential benefits of addressing these factors have been demonstrated in trials for other conditions, such as the PSY-HEART trial for coronary artery bypass graft surgery and the PSY-BREAST trial for breast cancer.^25^^,^^26^ Given the shared psychological risk factors associated with persistent gastrointestinal symptoms in IBS and UC, this trial includes both groups to identify disease-specific and transdiagnostic mechanisms. The SOMA.GUT trial evaluates whether a brief psychological expectation management intervention (GUT.EXPECT), targeting illness-related anxiety and dysfunctional symptom expectations, improves gastrointestinal symptoms when added to standard care in IBS and UC. To isolate its specific effects, a non-specific supportive intervention (GUT.SUPPORT) and a standard care-only group were included, resulting in a three-arm randomised controlled trial (RCT) design. The primary hypothesis was that an intervention targeting dysfunctional symptom expectations and illness-related anxiety would lead to a reduction in persistent gastrointestinal symptoms, as reflected by a change in the Irritable Bowel Syndrome—Severity Scoring System (IBS-SSS)^27^ from baseline to the 3-month follow-up. Secondary outcomes included the intervention's targets — illness-related anxiety and dysfunctional expectations — as well as symptom-related disability, psychosocial and inflammatory markers. Pre-specified subgroup analyses explored differential treatment responses by diagnosis, and patients evaluated the treatment.

## Methods

### Study design

The SOMA.GUT trial was an investigator-initiated, three-arm RCT to evaluate the effects of a mechanism-based intervention targeting dysfunctional symptom expectations and illness-related anxiety in patients with UC or IBS. The trial protocol has been published previously,^7^ and the trial was prospectively registered on 21st July 2021 (ISRCTN30800023). In addition to the mechanism-based intervention arm (GUT.EXPECT + standard care, SC), a second intervention arm (GUT.SUPPORT + SC) with the same treatment dose was included to give insights into non-specific psychological intervention effects, such as therapeutic alliance, attention, and time spent with a care provider. A standard care (SC) only group served as a control to assess the added effect of the interventions, resulting in a three-arm design. The trial was part of the Research Unit 5211 SOMACROSS, which investigates mechanisms underlying persistent somatic symptoms across ten medical conditions. Further details on this research unit, funded by the German Research Foundation (Deutsche Forschungsgemeinschaft, DFG) are available in the overall study protocol.^5^

Patients were involved through the national patient advocacy organisations for UC and IBS—the *German Crohn's and Colitis Association (DCCV e.V.)* and the *German IBS Self-Help Association (Deutsche Reizdarmselbsthilfe e.V.)*—in reviewing study materials, providing feedback on the intervention manual, supporting recruitment, and discussing findings during study meetings and a final patient day.

### Participants

The study was coordinated at the University Medical Centre Hamburg-Eppendorf, Germany. Given the online intervention format, nationwide recruitment was conducted through multiple channels, including the above-named patient advocacy organisations, outpatient clinics (University Medical Centre Hamburg-Eppendorf and Hamburg Israelitic Hospital), gastroenterological practices, social media, and public announcements (e.g., pharmacies, universities). Inclusion criteria were age ≥18 years; diagnosis of UC or IBS (Rome IV) confirmed by a medical report; moderate to severe gastrointestinal symptoms at screening according to the IBS—Severity Scoring System (IBS-SSS ≥175),^27^^,^^28^ treatment according to the current German guidelines,^29^^,^^30^ as verified by study investigators, and informed consent. Exclusion criteria were the need for acute emergency treatment, acute suicidality, psychotherapeutic treatment (past three months) and insufficient German language skills.

Patient recruitment followed a two-step procedure: First, patients completed an online screening to assess key eligibility criteria and, if eligible, submitted contact details. Second, the study team assessed detailed eligibility via telephone and also informed patients about the study. Upon verification of diagnosis through a medical report, eligible patients were invited to participate and provided written informed consent.

### Randomisation and masking

Patients were randomised 1:1:1 to one of three study arms, and randomisation was performed electronically using the web-based data collection system REDCap, stratified by diagnosis (UC or IBS) and gender. As data collection primarily relied on self-report online questionnaires, direct involvement of the study team was limited to providing data entry links. Blinding of study therapists was not feasible due to fidelity to treatment manuals. Patients were aware of their intervention status (control vs intervention) but were not informed whether they received the specific or non-specific intervention. To ensure treatment fidelity, selected therapy sessions from all study therapists were recorded and reviewed for adherence to the intervention manuals. As a manipulation check, patients in the intervention groups were asked at the 3-month follow-up what they perceived to be the focus of the intervention.

### Procedures

Both interventions (GUT.EXPECT and GUT.SUPPORT), described below, in the Appendix (p 2) and with further details on the underlying rationale in the study protocol,^7^ were matched for dose and therapist contact, each comprising four one-on-one 45-min videoconference sessions. The number of sessions builds on the successful change in expectations as realised by the PSY-HEART and PSY-BREAST trials, thus deemed sufficient for achieving change in the addressed mechanisms.^25^^,^^26^ The online delivery format was chosen to allow for nationwide recruitment, based on the existing evidence for online interventions in both conditions.^31^ The first three sessions were scheduled two weeks apart within the first six weeks followed by a booster session at three months. The manualised interventions were delivered individually by two psychologists, a gastroenterologist, and a physician in gastroenterology training, all of whom received extensive training and ongoing supervision by a licenced psychotherapist.

#### Standard care (SC)

Patients in all three trial arms continued to receive standard care from their usual care providers, e.g., gastroenterologists or general practitioners. The study team did not intervene in standard care procedures.

#### GUT.EXPECT + SC

The mechanism-based intervention GUT.EXPECT targeted dysfunctional symptom expectations and illness-related anxiety using cognitive-behavioural techniques. Adapted from the PSY-HEART^25^ and PSY-BREAST^26^ interventions, patients received a patient booklet, containing disease-specific information and worksheets to be used during the sessions and at home. In the first session, psychoeducation on the biopsychosocial model was provided, followed by an exploration of individual dysfunctional symptom expectations and illness-related anxiety. The second session involved cognitive restructuring of dysfunctional symptom expectations, followed by a guided gut imagery exercise, with an audio file for at home practice. In the third session, the vicious cycle of illness-related anxiety was explained and a behavioural experiment to disrupt this circle and resulting dysfunctional symptom expectations was prepared. In the final booster session, the implementation of this behavioural experiment was reviewed and the intervention content was consolidated. Throughout the intervention, patients were invited to build a personal ‘tool-box’ containing effective symptom management strategies.

#### GUT.SUPPORT + SC

In contrast, GUT.SUPPORT provided a non-specific, supportive intervention focussing on current stressful events and emotions. All sessions were reserved for topics raised by the patients, with the therapists instructed not to focus on illness-related anxiety and future-oriented dysfunctional symptom expectations, and to use an empathetic, validating approach. Unlike in GUT.EXPECT, no patient booklet was provided.

### Outcomes

The SOMA.GUT trial assessed outcomes at baseline, six weeks, three months (end of treatment, after the booster session), six months, and 12 months. Patients completed self-report questionnaires online via REDCap, a secure web application for data collection, at all time points. The primary outcome was the change in gastrointestinal symptom severity between baseline and 3-month follow-up, as measured by the Irritable Bowel Syndrome—Severity Scoring System (IBS-SSS, range 0–500).^27^

Secondary outcomes included changes in the targeted variables, namely illness-related anxiety (Whiteley Index short version, WI-7, range 0–7),^32^ and dysfunctional expectations, assessed via numerical rating scales (NRS, range 0–10) for expectations about symptom severity, symptom impairment, and symptom coping in six months each. In addition, other patient-relevant outcomes, including somatic symptom severity (Patient Health Questionnaire-15, PHQ-15, range 0–30),^33^ depression severity (Patient Health Questionnaire-9, PHQ-9, range 0–27),^34^ anxiety severity (Generalized Anxiety Disorder Scale-7, GAD-7, range 0–21),^35^ psychological distress related to somatic symptoms (Somatic Symptom Disorder—B Criteria Scale, SSD-12, range 0–48),^36^ symptom-related disability (Pain disability Index, PDI, range 0–70, adapted to general symptoms instead of pain only),^37^ and health-related quality of life (Short Form Health Survey, SF-12, range 0–100)^38^ were assessed. In patients with UC only, disease activity was assessed with the Simple Clinical Colitis Activity Index (SCCAI, range 0–19, active disease >5).^39^ Both patient groups were further characterised regarding somatic and mental comorbidities using the German version of the Self-administered Comorbidity Questionnaire (SCQ-D),^40^ complemented with 12 common mental disorders. The intervention evaluation included numerical rating scales on overall satisfaction, satisfaction with the number of sessions and the online format, the likelihood of recommending the intervention to a good friend, and questions on specific effects of the intervention.

Blood and stool samples were collected at baseline and 3-month follow-up to measure C-reactive protein (CRP) and faecal calprotectin, mostly sent by mail due to the nationwide recruitment. CRP (serum, detection limit ≥4 mg/l, normal <5 mg/l) and faecal calprotectin (stool, detection limit ≥5 μg/g, normal <50 μg/g) were analysed, with values below detection threshold imputed as half the detection limit.

### Choice of primary measure

The IBS-SSS as change from baseline to three months was chosen as the primary outcome to assess gastrointestinal symptom severity in both conditions. Originally developed for IBS, it captures abdominal pain, bloating, bowel function satisfaction, and symptom-related impairment over the last ten days, making it applicable for UC as well and allowing for comparison between both disorders. The IBS-SSS total score severity levels are defined as none (0–74), mild (75–174), moderate (175–299), and severe (300–500).^27^ The German version of the IBS-SSS has demonstrated high sensitivity to change.^28^

### Ethics

The study was approved by the Ethics Committee of the Hamburg Medical Association on 25th January 2021 (2020-10198-BO-ff) and conducted in accordance with the Declaration of Helsinki and Good Clinical Practice.

### Safety

Patient-reported adverse events, such as suicidality or hospital admission, were evaluated for necessary action and potential associations with the intervention. Serious adverse events (SAEs) and drop-outs were reviewed by the Data Safety and Monitoring Board (DSMB, composed of a psychosomatic specialist, a medical biologist and psychologist, a clinical psychologist, and a health psychologist) to determine appropriate handling, potential intervention-related causes, or patterns associated with specific study arms.

### Statistical analysis

The SOMA.GUT trial was powered with regard to the difference between GUT.EXPECT + SC vs the control condition (SC), primarily due to reasons of feasibility: With an expected very small difference in effect size between both intervention groups,^25^^,^^26^ the sample size would have accumulated to such a large sample size per group, that it would have exceeded the limits of both feasible recruitment and research budget. The study was not powered for comparisons between the two active interventions; therefore, this comparison was not conducted. However, an active comparator was included to gain exploratory insights into potential differences between a mechanism-based and an unspecific supportive intervention. Power calculation and expected loss to follow-up led to a planned total of n = 117 patients with UC and IBS, respectively, to be randomised (see study protocol for details).^7^

All analyses followed a pre-specified statistical analysis plan, registered with ISRCTN prior to database lock of the 3-month follow-up. Analyses were realised with R 4.3.2.^41^ Primary and secondary analyses were conducted according to the intention-to-treat principle, using the full analysis set. For the primary outcome, an analysis of covariance (ANCOVA) was conducted to investigate group differences in the change in IBS-SSS from baseline to the 3-month follow-up, adjusting for baseline IBS-SSS, underlying condition (UC vs IBS), and gender, in accordance with the randomisation scheme. In case of a significant difference between groups as indicated by the overall comparison F-statistic, pairwise comparisons were used to detect differences in efficacy between each treatment group as compared to SC in accordance to the closed testing principle. All secondary analyses were conducted as exploratory analyses, with p-values accordingly being reported as descriptive statistics only,^42^ while still providing an indication of the potential relevance of the observed effects.

As a sensitivity analysis for the primary endpoint, the potential influence of missing data — if exceeding 5% — was assessed by repeating the primary analysis using imputed data. Multiple imputation by chained equations (MICE), implemented via the *mice* package in R, was applied with 15 imputations. A longitudinal analysis of the primary outcome across all assessment points was conducted using a linear mixed-effects model with a random intercept for each participant, in which interactions between randomised group and time were allowed, and, in a sensitivity analysis, also between underlying condition, randomised group and time. Secondary outcomes were analysed according to their respective measurement scales. For metric outcomes, the same analytical approach as for the primary endpoint was applied. The underlying condition was excluded as a covariate in models assessing change in SCCAI, as this analysis was restricted to participants with UC. Pre-defined subgroup analyses were conducted to test for potential differential treatment effects in patient subgroups, as measured by the reduction in gastrointestinal symptom severity (IBS-SSS) between baseline and three months. Key subgroups included diagnosis (UC vs IBS) and gender (female vs male; diverse excluded from gender-specific analyses to protect confidentiality). Exploratory subgroups included age, migration background, education, duration of symptoms in years, UC disease activity, faecal calprotectin, CRP, and somatic comorbidities. Separate ANCOVA models were analysed in the intention-to-treat (ITT) population, adjusted for IBS-SSS at baseline and including randomised group allocation and the respective subgroup variable and its interaction term with group allocation as factors. Interaction test p-values and subgroup-specific means and pairwise effect differences with 95% confidence intervals were reported. The presented p-values were not adjusted for multiple comparisons. Post-hoc analyses were conducted for patients with IBS-SSS ≥175 at both screening (inclusion criterion) and baseline and for mental healthcare utilisation in all three trial arms over the 12-month study period.

### Role of the funding source

This study was carried out within the framework of Research Unit 5211 (RU 5211) ‘Persistent SOMAtic Symptoms ACROSS Diseases: From Risk Factors to Modification (SOMACROSS)’, funded by the German Research Foundation (Deutsche Forschungsgemeinschaft, DFG). The DFG grant numbers are LO 766/22-1 (BL) and LO 368/11-1 (AWL), see also https://gepris.dfg.de/gepris/projekt/460370451. The funder of the study had no role in the study design, data collection, data analysis, data interpretation, or writing of the report.

## Results

**Fig. 1:**
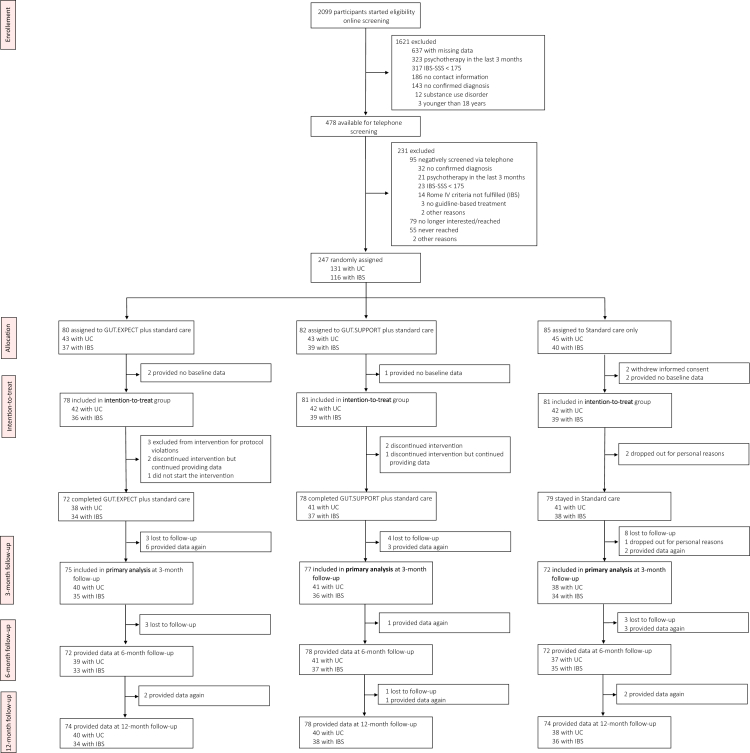
**Trial profile.** IBS-SSS, Irritable Bowel Syndrome–Symptom Severity System; IBS, Irritable Bowel Syndrome; UC, Ulcerative colitis; GUT.EXPECT, expectation management intervention in the SOMA.GUT-RCT; GUT.SUPPORT, unspecific supportive intervention in the SOMA.GUT-RCT.

Patient recruitment ran from 4 April 2022 to 24 February 2024 and ended upon reaching the target sample size. Key recruitment channels were patient advocacy organisations (*DCCV*, 38.1% of patients, *Deutsche Reizdarmselbsthilfe*, 15.8%). Of 2099 individuals screened online for eligibility, 1621 were excluded in this first step. Of the remaining 478, 231 were excluded after telephone assessment. Finally, a total of 247 patients were randomised: 131 with UC and 116 with IBS. Fig. 1 shows the detailed patient flow.

Five protocol violations occurred between randomisation and the start of the intervention: three patients started psychotherapy and were excluded from the intervention. Two patients initially screened positive regarding IBS-SSS ≥175, but dropped to a score <75 at baseline. These patients remained in the ITT analyses, with sensitivity analyses added for patients with IBS-SSS ≥175. Follow-up data were obtained from 215/240 (89.6%) of patients at six weeks, 221/240 (92.1%) at three months, 220/240 (91.7%) at six months, and 223/240 (92.9%) at 12 months.

Gender was reported as female by 176 participants (73.3%), male by 62 (25.8%), and diverse by two (0.8%). The mean age was 40.1 (SD 13.8) years. Mean baseline IBS-SSS gastrointestinal symptom severity was 268.6 (SD 92.9), indicating substantial symptom severity. Further sample characteristics by trial arm and diagnosis are shown in Table 1.

**Table 1:** Baseline characteristics of the three SOMA.GUT-RCT study arms in both diagnoses (IBS and UC).

	Ulcerative colitis (UC)			Irritable bowel syndrome (IBS)		
	Standard care (SC), N = 42	GUT.SUPPORT + SC, N = 42	GUT.EXPECT + SC, N = 42	Standard care (SC), N = 39	GUT.SUPPORT + SC, N = 39	GUT.EXPECT + SC, N = 36
Gender						
Female	30 (71.4%)	29 (69.0%)	31 (73.8%)	29 (74.4%)	29 (74.4%)	28 (77.8%)
Male	12 (28.6%)	12 (28.6%)	11 (26.2%)	10 (25.6%)	9 (23.1%)	8 (22.2%)
Diverse	0 (0.0%)	1 (2.4%)	0 (0.0%)	0 (0.0%)	1 (2.6%)	0 (0.0%)
Age	41.2 (11.5)	39.0 (12.8)	39.6 (12.7)	41.3 (15.1)	41.0 (16.2)	38.7 (14.2)
Migration background						
No	31 (73.8%)	40 (95.2%)	38 (90.5%)	31 (79.5%)	32 (82.1%)	31 (86.1%)
2nd Generation	9 (21.4%)	1 (2.4%)	4 (9.5%)	5 (12.8%)	5 (12.8%)	4 (11.1%)
1st Generation	2 (4.8%)	1 (2.4%)	0 (0.0%)	3 (7.7%)	2 (5.1%)	1 (2.8%)
Years of formal school education >10 years	32 (76.2%)	34 (81.0%)	32 (76.2%)	26 (66.7%)	33 (84.6%)	26 (72.2%)
Partnership [yes]	35 (83.3%)	33 (78.6%)	31 (73.8%)	27 (69.2%)	34 (87.2%)	26 (72.2%)
Occupational status						
Fully employed	13 (31.0%)	19 (45.2%)	17 (40.5%)	21 (53.8%)	14 (35.9%)	20 (55.6%)
Part-time employed	13 (31.0%)	12 (28.6%)	9 (21.4%)	9 (23.1%)	12 (30.8%)	5 (13.9%)
Retired, not employed or other	16 (38.0%)	11 (26.2%)	16 (38.0%)	9 (23.1%)	13 (33.3%)	11 (30.5%)
Currently unable to work/on sick leave	5 (11.9%)	3 (7.1%)	7 (16.6%)	2 (5.2%)	2 (5.1%)	5 (13.9%)
BMI (kg/m^2^)	26.2 (5.5)	24.8 (5.4)	24.1 (5.1)	23.8 (4.2)	23.7 (5.1)	22.7 (4.4)
No. of somatic comorbidities, SCQ-D soma	2.3 (1.4)	2.0 (1.4)	2.1 (1.5)	2.4 (1.4)	2.1 (1.2)	1.8 (1.1)
No. of mental comorbidities, SCQ-D psych	0.5 (0.9)	0.5 (0.8)	0.5 (1.0)	0.8 (1.2)	0.5 (0.6)	0.8 (1.6)
Currently prescribed medication [yes]	40 (95.2%)	40 (95.2%)	40 (95.2%)	19 (48.7%)	24 (61.5%)	15 (41.7%)
Current flare-up in UC (self-report), [Yes]	15 (35.7%)	22 (52.4%)	16 (38.1%)	/	/	/
Disease activity in UC, SCCAI	5.6 (2.8)	4.8 (2.4)	4.8 (2.7)	/	/	/
Faecal Calprotectin, μg/g	270.2 (619.1)	398.4 (1145.2)	201.4 (504.7)	7.5 (10.6)	6.9 (6.9)	15.4 (30.6)
C-reactive protein, mg/l	3.8 (4.4)	4.1 (5.7)	3.8 (4.0)	2.5 (2.0)	3.5 (4.0)	2.8 (3.0)
Gastrointestinal symptom severity, IBS-SSS	263.8 (89.6)	246.7 (92.3)	223.1 (85.9)	313.1 (81.1)	295.9 (94.3)	269.2 (114.2)
Time since onset of gastrointestinal symptoms, in years	10.9 (9.1)	10.7 (11.0)	9.3 (9.0)	6.7 (6.3)	7.2 (9.0)	8.1 (8.1)
Illness-related anxiety, WI-7	3.0 (1.8)	3.3 (1.9)	3.5 (1.9)	3.5 (1.8)	3.2 (1.9)	2.6 (1.9)
Depression severity, PHQ-9	8.9 (5.1)	8.7 (5.4)	8.9 (4.1)	8.4 (4.1)	7.9 (5.0)	7.9 (5.2)
Anxiety severity, GAD-7	6.5 (4.1)	7.3 (4.6)	6.3 (3.9)	7.2 (3.9)	7.4 (4.4)	6.9 (4.2)
Somatic symptom severity, PHQ-15	11.0 (4.1)	10.8 (3.8)	10.8 (4.9)	11.0 (3.9)	11.2 (3.2)	11.2 (4.4)
Psychological distress related to somatic symptoms, SSD-12	21.8 (9.7)	22.1 (10.2)	23.0 (8.0)	25.3 (7.9)	24.0 (8.9)	23.8 (7.9)
Symptom-related disability, PDI	31.4 (12.0)	27.1 (11.0)	31.6 (13.3)	32.1 (9.4)	31.2 (10.0)	29.9 (10.4)
Health-related quality of life, SF-12						
Physical component summary	41.7 (9.6)	42.3 (7.7)	40.5 (9.5)	41.8 (8.4)	44.1 (8.4)	46.0 (7.8)
Mental component summary	40.6 (12.0)	41.4 (11.3)	40.7 (9.4)	39.1 (9.8)	38.9 (10.8)	38.7 (11.0)

SC, Standard Care; GUT.EXPECT, expectation management intervention in the SOMA.GUT-RCT; GUT.SUPPORT, unspecific supportive intervention in the SOMA.GUT-RCT.

Data are shown as n/N (%), or mean (SD). BMI, Body Mass Index; SCQ-D, Self-reported Comorbidity Questionnaire—German version, complemented by 12 further mental disorders; UC, ulcerative colitis; IBS, irritable bowel syndrome; SCCAI, Simple Clinical Colitis Activity Index; IBS-SSS, Irritable Bowel Syndrome—Severity Scoring System; WI-7, Whiteley Index Short Form; PHQ-9, Patient Health Questionnaire-9; GAD-7, Generalized Anxiety Disorder Scale-7; PHQ-15, Patient Health Questionnaire-15; SSD-12, Somatic Symptom Disorder—B Criteria Scale-12; PDI, Pain Disability Index; used as symptom-related disability in this study; SF-12, Short-Form Health Survey-12.

CRP values under detection limit are imputed with half detection limit = 2.0 mg/l. Fecal calprotectin values under detection limit are imputed with half detection limit = 2.5 μg/g.

**Fig. 2:**
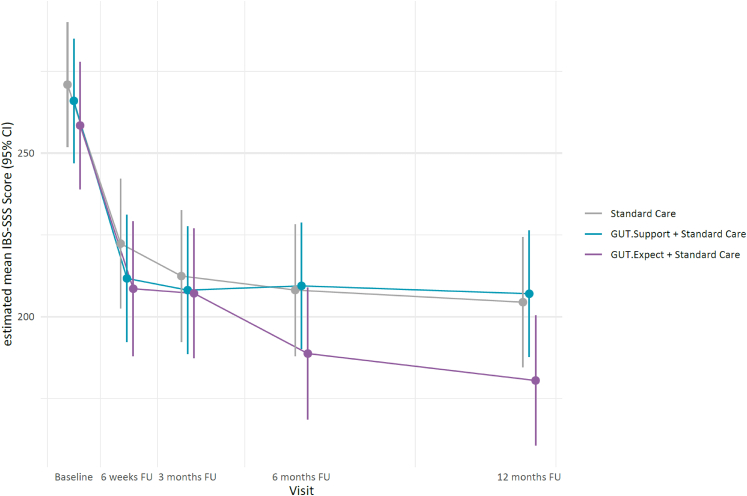
IBS-SSS scores over the course of the study in all three trial arms. Shown are estimated marginal means (dots) with pointwise 95% confidence intervals (vertical bars), derived from a linear mixed-effects model adjusted for baseline IBS-SSS, gender, and underlying condition. Visit is used as a categorical variable, the points are shifted horizontally for better visualisation. Lines connecting time points are for visual guidance only, as no data were collected between visits.

Gastrointestinal symptom severity (IBS-SSS) declined over time in all three study arms, with the greatest decline between baseline and six weeks (Fig. 2). The primary outcome, i.e., change in IBS-SSS gastrointestinal symptom severity between baseline and three months, was as follows: SC: −50.4 (95% CI −70.7 to −30.1), GUT.SUPPORT + SC: −55.4 (95% CI −75.0 to −35.9) and GUT.EXPECT + SC: −59.4 (95% CI −79.4 to −39.4). There were no significant differences between the three groups (global p = 0.83), nor in the exploratory pairwise comparisons between each intervention group compared to SC (Table 2). Post-hoc sensitivity analyses restricted to patients with IBS-SSS ≥175 at both screening and baseline (n = 181) yielded similar results (global p = 0.92). Analyses on the multiply imputed dataset confirmed the primary result (global p = 0.91; Appendix p 3).

**Table 2:** Results of the primary and secondary outcomes of the SOMA.GUT-RCT as change scores from baseline to 3-month follow-up.

	Marginal means				Pairwise comparisons			
	Standard care (SC)	GUT.SUPPORT + SC	GUT.EXPECT + SC	Global p-value (F-test)	GUT.SUPPORT + SC vs Standard care	p-value	GUT.EXPECT + SC vs Standard care	p-value
**Primary outcome**								
Gastrointestinal symptom severity, IBS-SSS	72; −50.4 (−70.7, −30.1)	77; −55.4 (−75.0, −35.9)	75; −59.4 (−79.4, −39.4)	0.83	−5.00 (−33.1, 23.1)	0.73	−8.99 (−37.7, 19.8)	0.54
**Secondary outcomes**								
Illness-related anxiety, WI-7	70; −0.2 (−0.5, 0.1)	77; −0.4 (−0.7, −0.1)	75; −0.8 (−1.1, −0.5)	0.02	−0.2 (−0.6, 0.3)	0.49	−0.6 (−1.1, −0.2)	0.01
Expectations about symptom severity in 6 months	71; −0.7 (−1.1, −0.2)	77; −0.9 (−1.2, −0.5)	75; −1.0 (−1.4, −0.6)	0.57	−0.2 (−0.8, 0.4)	0.50	−0.3 (−0.9, 0.3)	0.30
Expectations about impairment due to symptoms in 6 months	71; −0.7 (−1.2, −0.3)	77; −1.0 (−1.4, −0.6)	75; −0.8 (−1.3, −0.4)	0.69	−0.3 (−0.9, 0.3)	0.40	−0.1 (−0.7, 0.5)	0.79
Expectations about symptom coping in 6 months	71; −0.3 (−0.7, 0.2)	77; 0.8 (0.3, 1.2)	75; 1.0 (0.5, 1.4)	<0.001	1.0 (0.4, 1.7)	0.003	1.2 (0.5, 1.9)	<0.001
Somatic symptom severity, PHQ-15	72; −1.6 (−2.3, −0.9)	77; −1.1 (−1.8, −0.4)	75; −1.5 (−2.2, −0.8)	0.63	0.5 (−0.5, 1.5)	0.36	0.1 (−0.9, 1.1)	0.84
Disease activity, SCCAI (UC only)	40; −1.2 (−2.0, −0.4)	41; −0.7 (−1.4, 0.1)	41; −0.8 (−1.6, −0.1)	0.60	0.5 (−0.6, 1.6)	0.33	0.4 (−0.7, 1.5)	0.47
Faecal Calprotectin, (μg/g, log10)	66; −0.1 (−0.2, 0.1)	72; 0.0 (−0.2, 0.1)	72; 0.0 (−0.2, 0.1)	0.71	0.1 (−0.1, 0.3)	0.45	0.1 (−0.1, 0.3)	0.50
C-reactive protein, CRP (mg/l, log10)	67; 0.0 (−0.1, 0.1)	72; 0.0 (0.0, 0.1)	70; 0.0 (0.0, 0.1)	0.60	0.0 (−0.1, 0.1)	0.42	0.0 (0.0, 0.1)	0.35
Depression severity, PHQ-9	70; −0.6 (−1.4, 0.2)	76; −0.8 (−1.6, −0.1)	75; −2.1 (−2.8, −1.3)	0.01	−0.2 (−1.3, 0.9)	0.70	−1.5 (−2.6, −0.4)	0.01
Anxiety severity, GAD-7	70; −0.5 (−1.2, 0.1)	76; −0.7 (−1.3, −0.1)	75; −0.7 (−1.4, −0.1)	0.88	−0.2 (−1.1, 0.7)	0.72	−0.2 (−1.1, 0.7)	0.63
Psychological distress associated with somatic symptoms, SSD-12	71; −2.3 (−3.5, −1.0)	77; −1.8 (−3.0, −0.6)	75; −3.4 (−4.6, −2.1)	0.20	0.4 (−1.3, 2.2)	0.62	−1.1 (−2.8, 0.7)	0.22
SSD-12 cognitive subscale	71; −0.4 (−0.8, 0.1)	77; −0.1 (−0.5, 0.4)	75; −0.9 (−1.4, −0.5)	0.03	0.3 (−0.4, 0.9)	0.39	−0.6 (−1.2, 0.1)	0.09
SSD-12 affective subscale	71; −0.9 (−1.4, −0.4)	77; −0.8 (−1.3, −0.4)	75; −1.3 (−1.8, −0.8)	0.35	0.1 (−0.6, 0.8)	0.84	−0.4 (−1.1, 0.3)	0.26
SSD-12 behavioural subscale	71; −1.0 (−1.6, −0.4)	77; −0.9 (−1.5, −0.4)	75; −1.1 (−1.7, −0.5)	0.90	0.1 (−0.7, 0.9)	0.86	−0.1 (−0.9, 0.7)	0.77
Symptom-related disability, PDI	70; −0.7 (−3.2, 1.8)	77; −4.4 (−6.8, −2.0)	75; −5.8 (−8.2, −3.3)	0.02	−3.6 (−7.1, −0.2)	0.04	−5.0 (−8.5, −1.5)	0.01
Health-related quality of life, SF-12								
Physical component summary	70; 0.2 (−1.6, 1.9)	77; 1.7 (0.0, 3.3)	75; 2.0 (0.4, 3.7)	0.26	1.5 (−0.9, 3.9)	0.21	1.9 (−0.5, 4.3)	0.12
Mental component summary	70; 2.5 (0.6, 4.5)	77; 3.2 (1.3, 5.1)	75; 3.9 (2.0, 5.8)	0.63	0.6 (−2.1, 3.4)	0.64	1.3 (−1.4, 4.1)	0.33

Data are shown as n, estimated marginal mean changes from baseline (95% confidence intervals) and their differences (95% confidence intervals) compared to Standard care using the full analysis set (FAS).

SC, Standard Care; GUT.EXPECT, expectation management intervention in the SOMA.GUT-RCT; GUT.SUPPORT, unspecific supportive intervention in the SOMA.GUT-RCT. Change from baseline is reported in the respective units of the questionnaires. IBS-SSS, Irritable Bowel Syndrome—Severity Scoring System; PHQ-15, Patient Health Questionnaire-15; WI-7, Whiteley Index Short Form; PHQ-9, Patient Health Questionnaire-9; GAD-7, Generalized Anxiety Disorder Scale-7; SSD-12, Somatic Symptom Disorder—B Criteria Scale-12; PDI, Pain Disability Index, used as symptom-related disability in this study.

Fecal calprotectin values under detection limit are imputed with half detection limit = 2.5 μg/g. CRP values under detection limit are imputed with half detection limit = 2.0 mg/l.

In the following exploratory analyses (p-values used descriptively only), no relevant overall difference in IBS-SSS change was observed between groups at six months (Appendix pp 4 and 5) (global p = 0.21): SC only (−53.2; 95% CI −76.6 to −29.7), GUT.SUPPORT + SC (−53.5; 95% CI −75.9 to −31.1), and GUT.EXPECT + SC (−79.2; 95% CI −102.7 to −55.6). At 12 months (Appendix pp 6 and 7), however, the between-group differences in IBS-SSS change had widened in favour of the GUT.EXPECT + SC group (global p = 0.08): SC only (−55.3; 95% CI −77.9 to −32.7), GUT.SUPPORT + SC (−56.0; 95% CI −77.9 to −34.1) and GUT.EXPECT + SC (−87.7; 95% CI −110.4 to −65.0). A relevant but clinically marginal difference was observed in the pairwise comparison between GUT.EXPECT + SC and SC only (−32.4, 95% CI −64.7 to −0.1; p = 0.049).

Exploratory analyses of secondary outcomes showed no relevant group differences for most variables across follow-ups. However, several outcomes — particularly those targeted by GUT.EXPECT — differed meaningfully (Table 2, Appendix pp 4–7): At three months, there were differences in the reduction of illness-related anxiety, with the greatest reduction in the GUT.EXPECT + SC compared to SC only group. While expectations about symptom coping in six months’ time meaningfully improved in both intervention groups compared to SC, no relevant group differences emerged for expected symptom severity and expected symptom impairment. At six months, illness-related anxiety remained lower in GUT.EXPECT + SC compared to SC only. Symptom-related cognitions showed a relevant group difference in the comparison between GUT.EXPECT + SC vs SC. At 12 months, the intervention effects on the targeted variables had largely attenuated.

As for the other secondary outcomes, the decrease in depression severity differed between groups at three months, with the greatest improvement observed in the GUT.EXPECT + SC group. No relevant group differences were observed for the biomarkers faecal calprotectin and CRP (Table 2). For patients with UC, the SCCAI decreased in all three trial arms from baseline to three months with similar magnitude (see Table 2 and Appendix pp 4 and 6 for 6 and 12 months). Symptom-related disability (adapted PDI) decreased more in both intervention groups than in SC. However, these effects had largely subsided or disappeared by six and 12 months.

Regarding intervention evaluation at 3-month follow-up, overall satisfaction was high in both intervention groups. The likelihood of recommending this intervention to a good friend was 73% (95% CI 68%–79%) for GUT.SUPPORT, and 81% (95% CI 76%–87%) for GUT.EXPECT. Further evaluation (Table 3) showed high satisfaction with the online format and with the number of sessions. Regarding the manipulation check, i.e., the identification of the primary focus of their intervention, in GUT.SUPPORT, 95% selected “supportive conversations” across both diagnoses, while in GUT.EXPECT, 74% (UC) and 79% (IBS) selected “specific strategies for managing illness”.

**Table 3:** Results of the intervention evaluation in both intervention arms of the SOMA.GUT-RCT.

	Marginal means		Estimated mean difference
	GUT.SUPPORT + SC	GUT.EXPECT + SC	GUT.EXPECT + SC vs GUT.SUPPORT + SC
Satisfaction with the online treatment as a whole [0–4]	78; 3.2 (3.0, 3.4)	74; 3.4 (3.2, 3.6)	0.2 (−0.1, 0.5)
Satisfaction with the number of online sessions [0–4]	78; 2.7 (2.5, 2.9)	74; 3.0 (2.8, 3.2)	0.3 (0.0, 0.6)
Satisfaction with the delivery in online format [0–4]	78; 3.5 (3.3, 3.7)	74; 3.4 (3.2, 3.6)	0.0 (−0.3, 0.2)
The intervention			
has helped me to better understand my illness [0–4]	78; 2.3 (2.1, 2.5)	74; 3.0 (2.8, 3.2)	0.7 (0.4, 1.0)
has encouraged me to take action to improve my condition [0–4]	78; 2.9 (2.7, 3.1)	74; 3.3 (3.1, 3.5)	0.3 (0.1, 0.6)
has helped me to better deal with current stressful issues [0–4]	78; 2.9 (2.7, 3.1)	74; 3.1 (2.9, 3.3)	0.2 (−0.1, 0.4)
has helped me to reduce personal or professional stress [0–4]	78; 2.4 (2.2, 2.7)	74; 2.3 (2.1, 2.6)	−0.1 (−0.5, 0.2)
has helped me set helpful expectations about my condition [0–4]	78; 2.5 (2.3, 2.7)	74; 2.9 (2.7, 3.1)	0.4 (0.1, 0.7)
has helped to reduce my anxiety about my illness [0–4]	78; 2.3 (2.1, 2.5)	74; 2.7 (2.5, 2.9)	0.4 (0.1, 0.7)
How likely would you recommend this online treatment to a good friend with similar complaints? [0–10]	78; 7.3 (6.8, 7.9)	74; 8.1 (7.6, 8.7)	0.8 (0.0, 1.6)

Higher scores indicate higher levels of satisfaction. SC, Standard Care; GUT.EXPECT, expectation management intervention in the SOMA.GUT-RCT; GUT.SUPPORT, unspecific supportive intervention in the SOMA.GUT-RCT.

Exploratory subgroup analyses at three months revealed no relevant differences in treatment response by diagnosis (UC vs IBS), gender (male vs female), or further variables (Appendix pp 8–10). Exploratory analyses for the 12-month follow-up did not show relevant group differences in gastrointestinal symptom severity between patients with UC and those with IBS. Post-hoc analyses of the mental healthcare utilisation outside the study intervention across the three trial arms revealed no differences between trial arms (see Appendix p 11).

Nine serious adverse events (SAEs) occurred. These were seven hospitalisations due to UC or another medical condition (UC: 6, IBS: 1), one case of newly diagnosed cancer (IBS) and one case of passive suicidal thoughts (UC, SC group). SAEs were distributed across the treatment groups (SC: 2, GUT.SUPPORT + SC: 4 and GUT.EXPECT + SC: 3). No causal relationship of the SAEs with the interventions was identified by the study team or the DSMB.

## Discussion

In the three-arm randomised controlled SOMA.GUT trial, we evaluated a mechanism-based psychological intervention, grounded in a transdiagnostic biopsychosocial model,^6^ to test whether modifying illness-related anxiety and dysfunctional symptom expectations improves persistent gastrointestinal symptoms in UC and IBS. Contrary to our hypothesis, the mechanism-based intervention (GUT.EXPECT + SC) did not lead to a greater improvement in gastrointestinal symptoms at three months in the overall group comparison. Nevertheless, exploratory secondary analyses indicate potential modifications to the targets in the GUT.EXPECT intervention — illness-related anxiety and dysfunctional expectations — at three months, and showed a greater symptom improvement at 12 months beyond standard care, suggesting a delayed effect between mechanism change and symptom relief. Subgroup analyses revealed no difference in treatment efficacy on gastrointestinal symptoms between UC, IBS, or other patient characteristics. Most secondary outcomes were similar across groups, but GUT.EXPECT + SC led to greater reductions in depression severity and symptom-related disability at three months. Patient satisfaction was high for both interventions.

The delayed potential intervention benefits in gastrointestinal symptoms, occurring after rather than alongside changes in illness-related anxiety and dysfunctional symptom expectations, align with biopsychosocial models of persistent gastrointestinal symptoms^7^ and broader transdiagnostic models of somatic symptom persistence.^6^ Predictive coding models^6^ may offer a neurocognitive framework for understanding this temporal dissociation, as already investigated in symptoms such as pain.^43^ In this view, symptom expectations and illness-related anxiety act as prior beliefs that shape the interpretation of bodily sensations. While these priors can be modified through psychological intervention, the integration of updated expectations into perceptual processes and the resulting recalibration of symptom experience requires time. Reasons for the persistence of dysfunctional symptom expectations might stem from several sources, such as the assumed advantage of paying attention to a potentially dangerous and thus highly salient symptom or learnt associations in the sense of conditioned stimuli and symptoms.^43^ As the brain continues to test new priors against incoming sensory input, such prediction errors are gradually resolved, allowing the perceptual system to update symptom representations. This may explain why, in our study, improvements in psychological mechanisms preceded but did not immediately translate into changes in gastrointestinal symptom severity.

There may be several additional reasons for the non-superiority of GUT.EXPECT + SC in terms of gastrointestinal symptom severity at the primary assessment point at three months. Regression to the mean could have contributed to a substantial initial reduction in gastrointestinal symptoms in all three groups, especially since the entry criterion required an elevated symptom level. Additionally, gastrointestinal symptoms are known to fluctuate in both UC and IBS. Both factors applied equally to all three treatment groups due to randomisation and both diagnoses. Furthermore, although changes in the targeted variables were observed in GUT.EXPECT, the dosage of four sessions may have been insufficient to influence gastrointestinal symptoms promptly or to maintain changes in other outcomes. Psychological interventions showing benefits on somatic outcomes in IBS often involve six to 12 or even 30 sessions, though evidence on the necessity of higher doses remains mixed.^8^^,^^44^

The inclusion of both UC and IBS in the SOMA.GUT trial demonstrated the feasibility of investigating common and disease-specific mechanisms of symptom persistence within a single trial framework. Although we did not find relevant differences in treatment response between the two conditions, further studies, including those with larger sample sizes, are needed to thoroughly investigate the potential differences in response to mechanism-based treatments in these two conditions. This approach may help to improve future targeted psychological treatment strategies for chronic gastrointestinal disorders.

Evidence linking psychological risk factors to more severe symptoms provides further support for efforts to alleviate persistent symptoms in UC and IBS by targeting psychological factors.^23^ In SOMA.GUT, targeting only illness-related anxiety and dysfunctional symptom expectations may have been insufficient to modify gastrointestinal symptoms early in parallel with improvements in the two targeted psychological variables. While SOMA.GUT indicated effects on its psychological targets, symptom improvements were delayed, suggesting that broader, more intensive or personalised interventions may be required to achieve earlier and more intense symptomatic relief.

The biopsychosocial model of somatic symptom persistence proposes additional therapeutic targets for alleviating persistent somatic symptoms.^6^ Psychological factors such as depression influence prognosis, pain, and pain-related disability in patients with IBD and are well-established contributors to symptom persistence, as are high comorbidity rates in IBS.^19^^,^^45^^,^^46^ Psychological stress has been demonstrated to cause intestinal inflammation,^21^ and targeting stress as a mechanism might also be beneficial for IBS and IBD.^11^^,^^16^ Behavioural patterns such as all-or-nothing thinking or avoidance behaviour, which have shown promise in exposure-based therapy, were only indirectly addressed in the SOMA.GUT trial.^23^^,^^47^ Moreover, integrating psychological interventions with pharmacological, dietary, physical activity, or microbiome-based strategies may improve treatment efficacy. Taken together, a refined approach may involve optimising treatment dose, addressing multiple mechanisms simultaneously, or personalising interventions based on patient-specific biopsychosocial risk factors.^48^

The results of the SOMA.GUT trial should be interpreted in light of certain limitations. First, focussing on psychological mechanisms may have attracted participants with a greater openness to these interventions; a common selection bias in psychological trials that does not reduce the findings’ relevance for patients seeking such treatments. Second, blinding of patients and therapists was not possible, entailing potential bias, which is, however, common in psychotherapy research, and thus does not limit comparability with existing results. Third, the sample was predominantly female, and proficiency in the local language (German) was necessary to enable effective engagement with the treatment. While this may limit generalisability, it reflects the typical gender distribution in IBS and psychotherapy populations. Moreover, subgroup analyses revealed no differences in treatment efficacy by gender or migration background. Fourth, both interventions were delivered by the same four therapists, minimising therapist-related variability but increasing the possibility of spillover effects between treatments. Yet, the manipulation check confirmed distinct treatment focuses. Except for inflammation markers, outcomes were self-reported, which introduces the risk of social desirability effects. However, self-reported data remain essential for capturing subjective symptoms, and treatment effects and data collection method did not differ between groups. Finally, all secondary analyses were carried out as exploratory analyses, without adjustments for multiple comparisons. While these analyses provide relevant insights, their exploratory nature should be taken into account when interpreting the results. In addition to the above-mentioned suggestions for further developing the presented intervention approach, future studies on mechanism-based interventions should ideally consider a sample size large enough to allow for direct comparisons between specific and non-specific intervention approaches.

In conclusion, this trial indicates that illness-related anxiety and dysfunctional symptom expectations — key mechanisms in the biopsychosocial model of persistent somatic symptoms^5^ — can be modified through a mechanism-based psychological intervention in patients with IBS and UC. These changes were accompanied by improvements in other patient-relevant psychological outcomes, highlighting the clinical importance of the targeted mechanisms. However, early improvement in the primary outcome — gastrointestinal symptom severity at three months — was not achieved. Symptom improvement emerged only at 12 months, which might suggest a delayed translation of psychological change into somatic benefit. This dissociation points to a potential relevance of the hypothesised mechanisms, while also indicating that their modification alone may be insufficient for timely symptom relief. To achieve earlier and more pronounced clinical effects in IBS and UC, greater therapeutic intensity and broader biopsychosocial targeting may be required.

## Contributors

BL, AWL designed the study, with input from AZ, YN and VA. BL and AWL obtained the funding. KM and BL coordinated the study. KM, LP, AM, SHü, AW and BL performed the research and collected the data. Statistical analyses were performed by EV, PW and KS. KM and BL wrote the original draft of the manuscript. KM, LP accessed, EV, PW, KS and AZ verified the underlying data. All authors (KM, LP, SHü, AM, SH, AT, AZ, EV, PW, KS, VA, YN, AWL, BL) contributed to the interpretation of the data for the article and critically reviewed and edited the original draft. All authors (KM, LP, SHü, AM, SH, AT, AZ, EV, PW, KS, VA, YN, AWL, BL) approved the final version of the manuscript and take responsibility for the decision to submit for publication.

## Data sharing statement

Data can be requested from the corresponding author upon reasonable request. Data use and requests underlie data protection and the publication policy of the Research Unit SOMACROSS.

## Declaration of generative AI and AI-assisted technologies in the writing process

During the preparation of this work the authors used DeepL and ChatGPT to improve the readability and language of this article. After using these tools, the authors reviewed and edited the content as needed and take full responsibility for the content of the publication.

## Declaration of interests

SHu reports consultation fees from Janssen Cilag and AbbVie as well as honoraria for lectures and presentations from Janssen Cilag, Ferring, AbbVie, Falk, Sandoz/Hexal, Lilly, and BMS. AT reports research funding (no personal honoraria) from the German Research Foundation. She has received remunerations for a printed textbook from Ernst Reinhardt Publishing. VA reports consultation fees from Viatris and honoraria for lectures from Falk, Amedes, Repha, Luvos, and Medice. AWL reports research funding (no personal honoraria) from the German Research Foundation. He was Congress President of the 2023 Congress of the Deutsche Gesellschaft für Verdauungs-und Stoffwechselerkrankungen (DGVS) (unpaid) and is a board member of the Deutsche Gesellschaft für Verdauungs-und Stoffwechselerkrankungen (DGVS). BL reports research funding (no personal honoraria) from the German Research Foundation, the German Federal Ministry of Education and Research, the German Innovation Committee at the Joint Federal Committee, the European Commission's Horizon 2020 Framework Programme, the European Joint Programme for Rare Diseases (EJP), the Federal Ministry of Health, Germany, and the Foundation Psychosomatics of Spinal Diseases, Stuttgart, Germany. He received remunerations for several scientific book articles from various book publishers and as a committee member from Aarhus University, Denmark. He received travel expenses from the European Association of Psychosomatic Medicine (EAPM), and accommodation and meals from the Societatea de Medicina Biopsyhosociala, Romania. He received a travel grant for a lecture on the occasion of the presentation of the Alison Creed Award at the EAPM Conference in Lausanne, June 2024. He received remuneration and travel expenses for lectures at the Lindauer Psychotherapiewochen, April 2024, and the University Hospital Zurich, Department of Consultation-Liaison Psychiatry and Psychosomatic Medicine, March 2025. He is President of the German College of Psychosomatic Medicine (DKPM) (unpaid) since March 2024 and was a member of the Board of the European Association of Psychosomatic Medicine (EAPM) (unpaid) until 2022. He is member of the EIFFEL Study Oversight Committee (unpaid). All other authors (KM, LP, SHü, AM, AZ, EV, PW, KS, and YN) report no conflicts of interest.

## Appendix A Supplementary data

Supplement

Study Protocol
