# Supplementary material for: Efficacy of a mechanism-based psychological intervention for persistent gastrointestinal symptoms in ulcerative colitis and irritable bowel syndrome: results of a three-arm randomised controlled trial (SOMA.GUT-RCT)
Source: eClinicalMedicine. 2025 Nov 28;90:103663. doi: 10.1016/j.eclinm.2025.103663 (PMC12704308; doi:10.1016/j.eclinm.2025.103663)
Supplement: Supplement [file mmc1.pdf]

Supplementary material

Efficacy of a mechanism-based psychological intervention for persistent gastrointestinal symptoms in ulcerative colitis and irritable bowel syndrome: Results of a three-arm randomised controlled trial (SOMA.GUT-RCT)

Maehder et al.

Table of Contents

Figure S1	Structure and content of both intervention arms in the SOMA.GUT-RCT	page 2
Table S1	Results of the primary endpoint using multiply imputed data with m=15 imputations.	page 3
Table S2	Results of the primary and secondary outcomes as change scores from baseline to 6-month follow-up	page 4
Table S3	Results of the primary and secondary outcomes as change scores from baseline to 12-month follow-up	page 6
Table S4	Subgroup analyses of the SOMA.GUT-RCT for change in IBS-SSS from baseline to 3-month follow-up	page 8
Table S5	Utilisation of mental healthcare over the 12-month SOMA.GUT-RCT study period in all three trial arms (excluding the study interventions).	page 11
Figure S2	IBS-SSS scores over the course of the study in all three trial arms, stratified by underlying condition (UC vs. IBS)	page 12

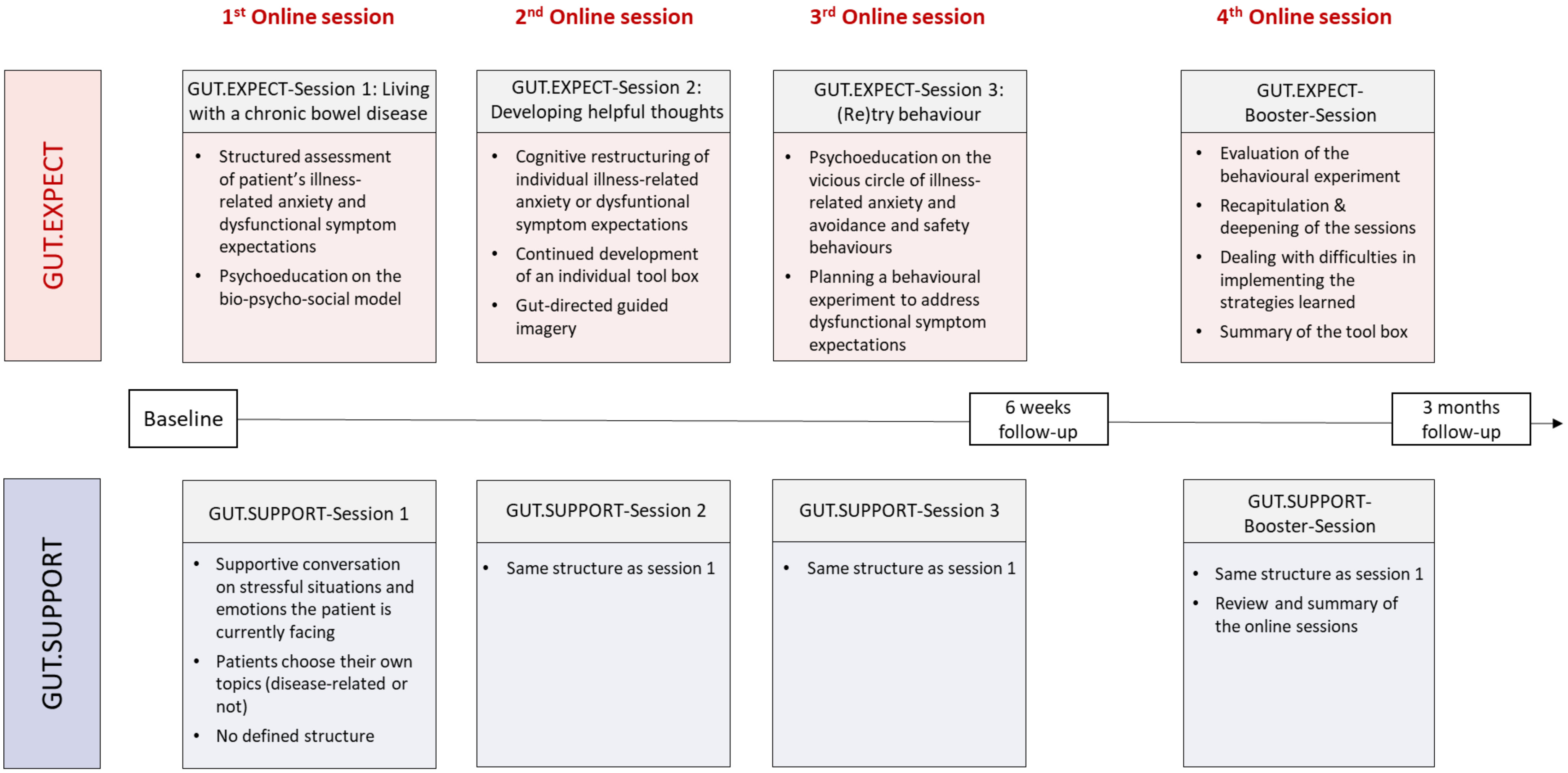

Supplement Figure S1: Structure and content of both intervention arms in the SOMA.GUT-RCT

	Marginal means				Pairwise comparisons			
	Standard Care (SC)	GUT.SUPPORT + SC	GUT.EXPECT + SC	Global p-value (F-test)	GUT.SUPPORT + SC vs. Standard care	p-value	GUT.EXPECT + SC vs. Standard care	p-value
**Primary outcome**								
Change in gastrointestinal symptom severity from baseline, IBS-SSS	-55·1 (-101·9, -8·4)	-57·5 (-101·0, -14·1)	-61·6 (-108·4, -14·9)	0·91	-2·4 (-31·4, 26·7)	0·87	-6·5 (-35·5, 22·5)	0·66

Data are shown as n, estimated marginal mean changes from baseline (95% confidence intervals) and their differences (95% confidence intervals) compared to Standard care using the full analysis set (FAS) with imputations.

SC = Standard Care. GUT.EXPECT = expectation management intervention in the SOMA.GUT-RCT. GUT.SUPPORT = unspecific supportive intervention in the SOMA.GUT-RCT. IBS-SSS = Irritable Bowel Syndrome – Symptom Severity Scale.

Supplement Table S1: Results of the primary endpoint using multiply imputed data with m=15 imputations.

Note: The proportion of missing values for the primary analysis was below 7%. Nevertheless, as it exceeded the pre-specified threshold, and as imputation-based analyses were only planned for the primary analysis, we repeated the primary analysis using imputed data as a sensitivity analysis. We included all baseline variables with an outflux coefficient (which is an indicator of the potential usefulness of a variable for imputing other variables) above 0.5 (according to van Buuren et al., 2008)[1]: i.e., age, gender, diagnosis and subsequently listed instruments. As predictors in our imputation model, we included randomised group and intermediate scores (IBS-SSS-Score, WI-7, SCQ-D, Expectations about symptom severity in 6 months, SSAS, PHQ-9, GAD-7, measured at baseline, 6 weeks, 3, 6 and 12 months).

[1] van Buuren, S. (2018). Flexible Imputation of Missing Data, Second Edition (2nd ed.). Chapman and Hall/CRC. https://doi.org/10.1201/9780429492259

	Marginal means				Pairwise comparisons			
	**Standard Care (SC)**	**GUT.SUPPORT + SC**	**GUT.EXPECT + SC**	**Global p-value (F-test)**	**GUT.SUPPORT + SC vs. Standard care**	**p-value**	**GUT.EXPECT + SC vs. Standard care**	**p-value**
**Primary outcome**								
Gastrointestinal symptom severity, IBS-SSS	72; -53·2 (-76·6, -29·7)	78; -53·5 (-75·9, -31·1)	72; -79·2 (-102·7, -55·6)	0·21	-0·3 (-32·7, 32·0)	0·99	-26·0 (-59·5, 7·4)	0·13
**Secondary outcomes**								
Illness-related anxiety, WI-7	72; -0·1 (-0·4, 0·2)	76; -0·2 (-0·5, 0·2)	72; -0·6 (-0·9, -0·3)	0·06	-0·1 (-0·5, 0·4)	0·79	-0·5 (-1·0, 0·0)	0·03
Expectations about symptom severity in 6 months	72; -1·0 (-1·4, -0·6)	76; -1·2 (-1·5, -0·8)	72; -1·3 (-1·6, -0·9)	0·61	-0·2 (-0·7, 0·4)	0·54	-0·3 (-0·8, 0·3)	0·33
Expectations about impairment due to symptoms in 6 months	72; -0·9 (-1·3, -0·5)	76; -1·1 (-1·5, -0·7)	72; -1·2 (-1·6, -0·8)	0·64	-0·2 (-0·8, 0·4)	0·55	-0·3 (-0·9, 0·3)	0·35
Expectations about symptom coping in 6 months	72; 0·3 (-0·2, 0·8)	76; 0·6 (0·1, 1·1)	72; 0·3 (-0·2, 0·8)	0·55	0·3 (-0·4, 1·0)	0·40	-0·1 (-0·8, 0·6)	0·86
Somatic symptom severity, PHQ-15	72; -2·1 (-2·9, 1·2)	78; -1·9 (-2·7, -1·1)	72; -2·0 (-2·8, -1·2)	0·98	0·1 (-1·0, 1·3)	0·82	0·0 (-1·1, 1·2)	0·94
Disease activity, SCCAI (UC only)	37; -1·4 (-2·0, -0·7)	41; -0·9 (-1·6, -0·3)	39; -1·3 (-2·0, -0·7)	0·59	0·4 (-0·5, 1·4)	0·38	0·0 (-1·0, 1·0)	0·98
Depression severity, PHQ-9	72; -0·9 (-1·8, -0·0)	76; -0·5 (-1·4, 0·3)	72; -1·9 (-2·8, -1·0)	0·07	0·4 (-0·8, 1·6)	0·53	-1·0 (-2·2, 0·2)	0·11
Anxiety severity, GAD-7	72; -0·8 (-1·6, 0·0)	76; -0·2 (-1·0, 0·5)	72; -0·8 (-1·6, 0·0)	0·57	0·5 (-0·6, 1·6)	0·36	0·0 (-1·1, 1·1)	1·00
	**Standard Care (SC)**	**GUT.SUPPORT + SC**	**GUT.EXPECT + SC**	**Global p-value (F-test)**	**GUT.SUPPORT + SC vs. Standard care**	**p-value**	**GUT.EXPECT + SC vs. Standard care**	**p-value**

	Marginal means				Pairwise comparisons			
Psychological distress associated with somatic symptoms, SSD-12	72; -2·5<br>(-4·0, -1·0)	78; -2·0<br>(-3·5, -0·6)	72; -4·2<br>(-5·7, -2·7)	0·11	0·5<br>(-1·6, 2·6)	0·64	-1·7<br>(-3·8, 0·5)	0·13
SSD-12 cognitive subscale	72; -0·3<br>(-0·8, 0·2)	78; -0·2<br>(-0·6, 0·3)	72; -1·1<br>(-1·6, -0·6)	0·02	0·2<br>(-0·5, 0·9)	0·63	-0·8<br>(-1·5, -0·1)	0·03
SSD-12 affective subscale	72; -1·1<br>(-1·8, -0·5)	78; -0·9<br>(-1·4, -0·3)	72; -1·5<br>(-2·1, -0·9)	0·29	0·3<br>(-0·6, 1·1)	0·49	-0·4<br>(-1·2, 0·5)	0·39
SSD-12 behavioral subscale	72; -1·0<br>(-1·7, -0·4)	78; -1·0<br>(-1·7, -0·4)	72; -1·5<br>(-2·2, -0·8)	0·52	0·0<br>(-0·9, 1·0)	0·99	-0·5<br>(-1·5, 0·5)	0·33
Symptom-related disability, PDI	72; -3·2<br>(-5·7, -0·7)	76; -4·3<br>(-6·8, -1·9)	72; -6·5<br>(-8·9, -4·0)	0·18	-1·2<br>(-4·7, 2·3)	0·51	-3·3<br>(-6·8, 0·2)	0·07
Health-related quality of life, SF-12								
Physical component summary	72; 1·1<br>(-0·5, 2·8)	76; 2·0<br>(0·5, 3·6)	72; 2·9<br>(1·3, 4·5)	0·32	0·9<br>(-1·4, 3·2)	0·44	1·8<br>(-0·5, 4·1)	0·13
Mental component summary	72; 2·3<br>(0·2, 4·5)	76; 1·1<br>(-1·0, 3·2)	72; 2·0<br>(-0·1, 4·2)	0·68	-1·2<br>(-4·2, 1·7)	0·41	-0·3<br>(-3·3, 2·7)	0·84

Data are shown as n, estimated marginal means (95% confidence intervals) and their differences (95% confidence intervals) compared to Standard care using the full analysis set (FAS).

SC = Standard Care. GUT.EXPECT = expectation management intervention in the SOMA.GUT-RCT. GUT.SUPPORT = unspecific supportive intervention in the SOMA.GUT-RCT. IBS-SSS = Irritable Bowel Syndrome – Symptom Severity Scale. PHQ-15 = Patient Health Questionnaire-15. WI-7 = Whiteley Index-7. PHQ-9 = Patient Health Questionnaire-9. GAD-7 = Generalized Anxiety Disorder Scale-7. SSD-12 = Somatic Symptom Disorder – B Criteria Scale-12. PDI = Pain Disability Index, used as symptom-related disability in this study.

Supplement Table S2: Results of the primary and secondary outcomes as change scores from baseline to 6-month follow-up

	Marginal means				Pairwise comparisons			
	Standard Care (SC)	GUT.SUPPORT + SC	GUT.EXPECT + SC	Global p-value (F-test)	GUT.SUPPORT + SC vs. Standard care	p-value	GUT.EXPECT + SC vs. Standard care	p-value
**Primary outcome**								
Gastrointestinal symptom severity, IBS-SSS	74; -55·3 (-77·9,-32·7)	78; -56·0 (-77·9, -34·1)	74; -87·7 (-110·4, -65·0)	0·08	-0·7 (-32·1, 30·8)	0·97	-32·4 (-64·7, -0·1)	0·049
**Secondary outcomes**								
Illness-related anxiety, WI-7	74; -0·5 (-0·8, -0·1)	77; -0·4 (-0·8, -0·1)	74; -0·7 (-1·1, -0·4)	0·43	0·0 (-0·4, 0·5)	0·86	-0·3 (-0·8, 0·2)	0·31
Expectations about symptom severity in 6 months	74; -0·9 (-1·4, -0·4)	77; -1·0 (-1·4, -0·5)	74; -1·3 (-1·8, -0·9)	0·38	-0·1 (-0·7, 0·6)	0·79	-0·4 (-1·1, 0·2)	0·19
Expectations about impairment due, symptoms in 6 months	74; -1·0 (-1·5, -0·5)	77; -1·0 (-1·5, -0·6)	74; -1·4 (-1·9, -1·0)	0·36	-0·1 (-0·7, 0·6)	0·84	-0·5 (-1·1, 0·2)	0·19
Expectations about symptom coping in 6 months	74; 0·4 (-0·2, 0·9)	77; 0·0 (-0·5, 0·5)	74; 0·7 (0·2, 1·2)	0·19	-0·4 (-1·1, 0·4)	0·35	0·3 (-0·4, 1·1)	0·38
Somatic symptom severity, PHQ-15	74; -2·1 (-3·0, -1·3)	78; -1·4 (-2·3, -0·6)	74; -2·0 (-2·8, -1·2)	0·47	0·7 (-0·5, 1·9)	0·25	0·1 (-1·1, 1·3)	0·84
Disease activity, SCCAI (UC only)	39; -0·9 (-1·7, -0·1)	41; -1·5 (-2·3, -0·7)	40; -1·4 (-2·2, -0·6)	0·49	-0·6 (-1·8, 0·5)	0·28	-0·6 (-1·7, 0·6)	0·33
Depression severity, PHQ-9	74; -0·2 (-1·0, 0·6)	77; -0·6 (-1·4, 0·2)	74; -1·5 (-2·3, -0·7)	0·08	-0·4 (-1·5, 0·7)	0·49	-1·3 (-2·4, -0·1)	0·03
Anxiety severity, GAD-7	74; -0·6 (-1·3, 0·2)	77; -0·9 (-1·7, -0·2)	74; -0·7 (-1·5, 0·0)	0·79	-0·4 (-1·4, 0·7)	0·50	-0·1 (-1·2, 0·9)	0·81
Psychological distress associated with somatic symptoms, SSD-12	74; -3·3 (-5·0, -1·6)	78; -2·5 (-4·2, -0·9)	74; -4·9 (-6·6, -3·2)	0·13	0·8 (-1·6, 3·2)	0·51	-1·6 (-4·0, 0·8)	0·19

	Marginal means				Pairwise comparisons			
	**Standard Care (SC)**	**GUT.SUPPORT + SC**	**GUT.EXPECT + SC**	**Global p-value (F-test)**	**GUT.SUPPORT + SC vs. Standard care**	**p-value**	**GUT.EXPECT + SC vs. Standard care**	**p-value**
SSD-12 cognitive subscale	74; -0·5 (-1·0, 0·0)	78; -0·3 (-0·8, 0·2)	74; -1·2 (-1·7, -0·6)	0·06	0·2 (-0·5, 1·0)	0·58	-0·7 (-1·4, 0·1)	0·09
SSD-12 affective subscale	74; -1·6 (-2·3, -0·9)	78; -1·0 (-1·7, -0·3)	74; -2·0 (-2·7, -1·2)	0·14	0·6 (-0·4, 1·6)	0·22	-0·4 (-1·4, 0·6)	0·46
SSD-12 behavioral subscale	74; -1·3 (-2·0, -0·6)	78; -1·3 (-2·0, -0·6)	74; -1·8 (-2·5, -1·1)	0·49	-0·1 (-1·0, 0·9)	0·89	-0·6 (-1·5, 0·4)	0·27
Symptom-related disability, PDI	74; -4·6 (-7·2, -2·0)	77; -5·6 (-8·1, -3·1)	74; -8·0 (-10·5, -5·4)	0·17	-1·0 (-4·6, 2·6)	0·58	-3·4 (-7·0, 0·3)	0·07
Health-related quality of life, SF-12								
Physical component summary	74; 0·7 (-1·0, 2·4)	77; 2·4 (0·7, 4·0)	74; 3·4 (1·7, 5·0)	0·08	1·7 (-0·7, 4·0)	0·16	2·7 (0·3, 5·0)	0·03
Mental component summary	74; 2·3 (0·2, 4·5)	77; 2·7 (0·6, 4·8)	74; 2·4 (0·2, 4·5)	0·97	0·4 (-2·6, 3·4)	0·82	0·0 (-3·0, 3·0)	0·99

Data are shown as n, estimated marginal mean changes from baseline (95% confidence intervals) and their differences (95% confidence intervals) compared to Standard care using the full analysis set (FAS).

SC = Standard Care. GUT.EXPECT = expectation management intervention in the SOMA.GUT-RCT. GUT.SUPPORT = unspecific supportive intervention in the SOMA.GUT-RCT. IBS-SSS = Irritable Bowel Syndrome – Symptom Severity Scale. PHQ-15 = Patient Health Questionnaire-15. WI-7 = Whiteley Index-7. PHQ-9 = Patient Health Questionnaire-9. GAD-7 = Generalized Anxiety Disorder Scale-7. SSD-12 = Somatic Symptom Disorder – B Criteria Scale-12. PDI = Pain Disability Index, used as symptom-related disability in this study.

Supplement Table S3: Results of the primary and secondary outcomes as change scores from baseline to 12-month follow-up

	Change in IBS-SSS in the three trial arms			Pairwise comparisons						Overall comparison	
	**Standard Care (SC)**	**GUT. SUPPORT + SC**	**GUT. EXPECT + SC**	**GUT. SUPPORT + SC vs. SC**	**p-value**	**GUT. EXPECT + SC vs. SC**	**p-value**	**GUT.EXPECT + SC vs. GUT.SUPPORT + SC**	**p-value**	**Sub-group F-test**	**Inter-action p-value**
**Diagnosis**											
Ulcerative colitis (UC)	-66·3 (-94·6, -38·0)	-67·3 (-94·7, -40·0)	-49·9 (-78·1, -21·8)	-1·0 (-40·3, 38·3)	0·96	16·4 (-23·5, 56·3)	0·42	17·4 (-21·5, 56·3)	0·38	0·62	0·18
Irritable Bowel Syndrome (IBS)	-35·5 (-65·7, -5·2)	-41·5 (-70·9, -12·1)	-67·8 (-97·2, -38·3)	-6·0 (-47·7, 35·6)	0·78	-32·3 (-74·5, 9·9)	0·13	-26·3 (-67·9, 15·3)	0·22	0·28	
**Gender**											
Male	-75·0 (-112·7, -37·3)	-97·8 (-136·4, -59·1)	-84·4 (-125·1, -43·7)	-22·8 (-76·7, 31·2)	0·41	-9·4 (-64·9, 46·1)	0·74	13·4 (-43·0, 69·7)	0·64	0·71	0·71
Female	-42·1 (-66·4, -17·8)	-40·4 (-63·7, -17·1)	-49·4 (-72·5, -26·3)	1·7 (-31·9, 35·3)	0·92	-7·3 (-40·9, 26·3)	0·67	-9·0 (-41·8, 23·8)	0·59	0·85	
**Age [divided in terciles]**											
Young [18·9,31·7]	-69·1 (-107·8, -30·4)	-35·0 (-67·8, -2·2)	-70·4 (-104·0, -36·9)	34·1 (-16·7, 84·8)	0·19	-1·3 (-52·6, 49·9)	0·96	-35·4 (-82·5, 11·7)	0·14	0·26	
Middle aged [31·7,43·7]	-47·6 (-81·6, -13·6)	-100·7 (-139·5, -61·9)	-39·1 (-73·4, -4·8)	-53·1 (-104·7, -1·4)	0·04	8·5 (-39·9, 57·0)	0·73	61·6 (10·0, 113·2)	0·02	0·05	0·05
Old [43·7,79·9]	-42·6 (-76·8, -8·4)	-43·5 (-75·7, -11·3)	-65·9 (-102·8, -29·0)	-0·9 (-47·7, 45·9)	0·97	-23·3 (-73·6, 27·0)	0·36	-22·4 (-71·4, 26·6)	0·37	0·59	
**Migration background**											
No migration background	-59·1 (-82·4, -35·8)	-59·6 (-80·7, -38·5)	-56·1 (-77·6, -34·5)	-0·5 (-31·8, 30·8)	0·97	3·0 (-28·9, 34·9)	0·85	3·5 (-26·8, 33·8)	0·82	0·97	
2nd Generation	-58·1 (-108·5, -7·6)	-8·2 (-79·1, 62·8)	-59·5 (-120·9, 2·0)	49·9 (-36·9, 136·7)	0·26	-1·4 (-81·2, 78·4)	0·97	-51·3 (-145·3, 42·7)	0·28	0·47	0·14
1st Generation	60·2 (-26·8, 147·3)	-57·1 (-158·6, 44·4)	-134·6 (-308·2, 38·9)	-117·3 (-250·2, 15·5)	0·08	-194·8 (-389·1, -0·6)	0·05	-77·5 (-278·7, 123·7)	0·45	0·07	
**Education years**											

	Change in IBS-SSS in the three trial arms			Pairwise comparisons						Overall comparison	
	**Standard Care (SC)**	**GUT. SUPPORT + SC**	**GUT. EXPECT + SC**	**GUT. SUPPORT + SC vs. SC**	**p-value**	**GUT. EXPECT + SC vs. SC**	**p-value**	**GUT.EXPECT + SC vs. GUT.SUPPORT + SC**	**p-value**	**Sub-group F-test**	**Inter-action p-value**
≤ 10 years in school	-37·8 (-76·4, 0·7)	-40·6 (-89·4, 8·1)	-49·0 (-89·3, -8·7)	-2·8 (-65·0, 59·4)	0·93	-11·1 (-67·0, 44·7)	0·70	-8·3 (-71·5, 54·8)	0·80	0·92	0·97
> 10 years in school	-57·8 (-82·4, -33·2)	-58·4 (-80·4, -36·4)	-60·9 (-84·6, -37·2)	-0·5 (-33·5, 32·4)	0·97	-3·1 (-37·4, 31·2)	0·86	-2·6 (-35·1, 29·9)	0·88	0·98	
**Duration of symptoms**											
< 5 years	-46·7 (-80·8, -12·5)	-68·9 (-99·2, -38·6)	-73·6 (-103·1, -44·0)	-22·3 (-67·9, 23·4)	0·34	-26·9 (-72·1, 18·3)	0·24	-4·6 (-46·9, 37·7)	0·83	0·47	
5-10 years	-32·4 (-68·8, 3·9)	-27·0 (-68·3, 14·3)	-59·3 (-103·1, -15·5)	5·4 (-49·4, 60·2)	0·85	-26·9 (-84·0, 30·2)	0·35	-32·3 (-92·9, 28·3)	0·29	0·53	0·25
> 10 years	-77·2 (-113·6, -40·8)	-57·6 (-91·7, -23·5)	-34·8 (-70·3, 0·8)	19·5 (-30·4, 69·5)	0·44	42·4 (-8·6, 93·4)	0·10	22·9 (-26·4, 72·1)	0·36	0·26	
**Disease activity (UC only)**											
SCCAI <5	-56·1 (-101·7, -10·5)	-38·4 (-79·7, 2·8)	-56·0 (-96·5, -15·6)	17·6 (-43·2, 78·5)	0·57	0·0 (-60·0, 60·0)	1·0	-17·6 (-74·8, 39·6)	0·54	0·79	0·29
SCCAI ≥5	-58·8 (-96·9, -20·7)	-75·8 (-112·4, -39·2)	-31·3 (-69·3, 6·7)	-17·0 (-68·6, 34·7)	0·52	27·5 (-26·7, 81·7)	0·32	44·5 (-8·5, 97·4)	0·10	0·25	
**Baseline IBS-SSS score**											
mild [≥75-174]	17·8 (-40·6, 76·2)	2·9 (-44·0, 49·7)	-8·3 (-49·6, 33·0)	-14·9 (-89·8, 60·0)	0·70	-26·1 (-97·7, 45·4)	0·47	-11·2 (-73·6, 51·3)	0·72	0·77	
moderate [≥175 - 300]	-64·2 (-93·4, -35·0)	-53·5 (-83·6, -23·5)	-46·2 (-74·2, -18·1)	10·6 (-31·3, 52·5)	0·62	18·0 (-22·5, 58·5)	0·38	7·4 (-33·7, 48·5)	0·72	0·68	0·51
severe [>300]	-73·3 (-107·1, -39·6)	-92·1 (-124·6, -59·5)	-115·0 (-158·8, -71·2)	-18·7 (-65·6, 28·1)	0·43	-41·7 (-97·0, 13·6)	0·14	-22·9 (-77·5, 31·6)	0·41	0·33	
**Faecal calprotectin (FCAL)**											

	Change in IBS-SSS in the three trial arms			Pairwise comparisons						Overall comparison	
	Standard Care (SC)	GUT. SUPPORT + SC	GUT. EXPECT + SC	GUT. SUPPORT + SC vs. SC	p-value	GUT. EXPECT + SC vs. SC	p-value	GUT.EXPECT + SC vs. GUT.SUPPORT + SC	p-value	Sub-group F-test	Inter-action p-value
FCAL <50	-46·6 (-82·0, -11·1)	-33·6 (-69·1, 1·9)	-52·9 (-88·5, -17·3)	13·0 (-37·1, 63·2)	0·61	-6·3 (-56·5, 44·0)	0·81	-19·3 (-69·3, 30·7)	0·45	0·74	0·10
FCAL ≥50	-89·7 (-140·9, -38·5)	-83·6 (-126·1, -41·0)	-24·3 (-68·2, 19·7)	6·1 (-60·1, 72·2)	0·86	65·4 (-1·7, 132·6)	0·06	59·3 (-1·8, 120·5)	0·06	0·09	
**Systemic inflammation**											
CRP <5	-48·5 (-71·8, -25·2)	-54·2 (-77·0, -31·4)	-56·2 (-78·4, -33·9)	-5·7 (-38·3, 26·8)	0·73	-7·7 (-40·1, 24·7)	0·64	-2·0 (-33·8, 29·9)	0·90	0·89	0·95
CRP ≥5	-54·2 (-107·5, -1·0)	-54·2 (-103·3, -5·0)	-69·7 (-125·6, -13·9)	0·1 (-72·2, 72·4)	1·0	-15·5 (-92·7, 61·7)	0·69	-15·6 (-90·1, 58·9)	0·68	0·90	
**Number of somatic comorbidities**											
SCQ-D soma <2	-88·5 (-156·8, -20·3)	-148·5 (-223·2, -73·7)	-58·6 (-119·6, 2·3)	-59·9 (-161·1, 41·2)	0·24	29·9 (-61·1, 120·9)	0·52	89·8 (-6·3, 186·0)	0·07	0·18	
SCQ-D soma 2-3	-81·8 (-120·2, -43·5)	-28·1 (-58·2, 1·9)	-98·4 (-129·0, -67·9)	53·7 (5·0, 102·4)	0·03	-16·6 (-65·7, 32·5)	0·51	-70·3 (-113·2, -27·4)	0·001	0·004	< 0·001
SCQ-D soma ≥4	-34·8 (-59·4, -10·3)	-64·4 (-90·5, -38·2)	-25·7 (-53·2, 1·8)	-29·5 (-65·3, 6·2)	0·11	9·1 (-27·8, 46·0)	0·63	38·6 (0·7, 76·6)	0·05	0·11	

Prespecified subgroup analyses for change in IBS-SSS from baseline to 3-month follow-up in both intervention arms compared to control group. Data are shown as estimated marginal mean changes from baseline (95% confidence intervals) and their differences (95% confidence intervals) compared to Standard care using the full analysis set (FAS).

IBS-SSS = Irritable Bowel Syndrome – Symptom Severity Scale. SC = Standard Care. GUT.EXPECT = expectation management intervention in the SOMA.GUT-RCT. GUT.SUPPORT = unspecific supportive intervention in the SOMA.GUT-RCT. UC = Ulcerative colitis. IBS = Irritable Bowel Syndrome. Migration background 2nd generation = one or both of parents are not born in Germany. Migration background 1st generation = Patient is not born in Germany. SCCAI = Simple Clinical Colitis Activity Index. CRP = C-reactive protein. SCQ-D soma = Self-reported Comorbidity Questionnaire – German version, somatic conditions only.

Supplement Table S4: Subgroup analyses of the SOMA.GUT-RCT for change in IBS-SSS from baseline to 3-month follow-up

	Trial arm allocation					
	**Standard Care (SC), N = 74**	**GUT.SUPPORT + SC, N = 77**	**GUT.EXPECT + SC, N = 74**	***χ2***	***df***	***p***
**Outpatient psychotherapeutic treatment[1]**						
Yes	9 (12·2)	16 (20·8)	11 (14·9)	2·19	2	0·334
No	65 (87·8)	61 (79·2)	63 (85·1)			
Frequency (M (SD))	13·6 (16·6)	13·3 (15·4)	9·8 (8·8)			
**Outpatient psychiatric treatment[2]**						
Yes	2 (2·7)	5 (6·5)	5 (6·8)	1·52	2	0·469
No	72 (97·3)	72 (93·5)	69 (93·2)			
Frequency (M (SD))	3·0 (1·4)	4·4 (3·3)	4·0 (2·4)			
**Psychosomatic treatment[3]**						
Yes	1 (1·4)	2 (2·6)	3 (4·1)	1·04	2	0·593
No	73 (98·6)	75 (97·4)	71 (95·9)			
Frequency (M (SD))	2·0	42·0 (31·1)	4·3 (1·5)			

Note: All psychotherapeutic treatments listed took place after the intervention in the two intervention arms. There were no significant differences between the two bowel conditions regarding use of the different types of services. Data is shown as n (%) or M (SD); M = Mean, SD = Standard deviation.

[1] n = 9 reported IBS or UC as the reason for outpatient psychotherapeutic treatment.

[2] Medical treatment by a psychiatrist, usually including pharmacotherapy; n = 1 reported IBS or UC as the reason for outpatient psychiatric treatment.

[3] Inpatient (n = 2), day clinic (n = 1) or outpatient (n = 3) treatment in a psychosomatic medicine facility, combining medical and psychotherapeutic approaches; n = 4 reported IBS or UC as the reason for psychosomatic treatment.

Supplement Table S5. Utilisation of mental healthcare over the 12-month SOMA.GUT-RCT study period in all three trial arms (excluding the study interventions).

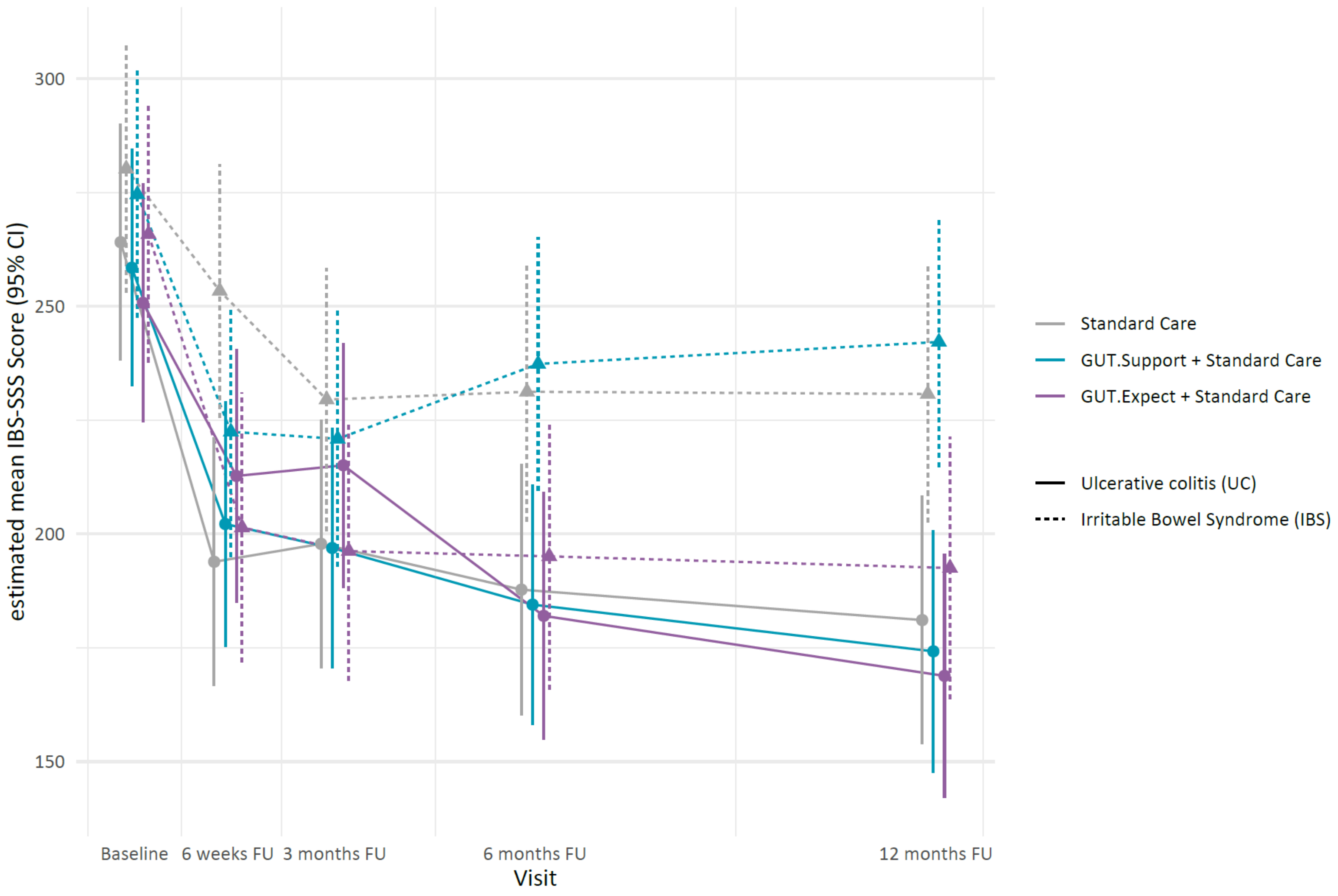

Supplement Figure S2: IBS-SSS scores over the course of the study in all three trial arms, stratified by underlying condition (UC vs IBS). Shown are estimated marginal means (dots) with pointwise 95% confidence intervals (vertical bars), derived from a linear mixed-effects model adjusted for baseline IBS-SSS, gender, and underlying condition. Visit is used as a categorical variable, the points are shifted horizontally for better visualisation. Lines connecting time points are for visual guidance only, as no data were collected between visits.
